# Social Influence in Australian University Institutional Eating: A Qualitative Content Analysis

**DOI:** 10.1002/hpja.70039

**Published:** 2025-05-13

**Authors:** Katrina A. Pitman, Julia Carins, Sharyn Rundle‐Thiele, Lisa Vincze

**Affiliations:** ^1^ Griffith Business School, Department of Marketing, Social Marketing @ Griffith Griffith University Brisbane Queensland Australia; ^2^ School of Health Science and Social Work Griffith University Gold Coast Queensland Australia

**Keywords:** eating behaviour, health, institutional, nutrition, social influence, socialisation

## Abstract

**Introduction:**

Social influence has been recognised as a primary determinant of eating behaviour, and an effective behavioural change mechanism. Institutional settings offer a conducive environment for eating behaviour research, and few have examined multiple social influences interacting within an Australian institutional eating setting. Responding to calls to improve the use and reporting of theory within social marketing research, this study aimed to explore which social influences within the Consumer Socialisation Theory (CST) were evident in an institutional eating environment, to determine if the use of the CST could advance theory use within social marketing and behavioural change research.

**Methods:**

Semi‐structured interviews (*n* = 16) were conducted with students residing in university college accommodation with provided meals. A deductive content analysis method was used to identify the CST social influences of interaction, modelling, social norms and reinforcement.

**Results:**

All theory defined social influences were evident. While all four social influences were present, participants identified interaction, modelling and social norms as having a greater influence within this context. The CST was successfully applied and helped to identify the different social influences within this study.

**Conclusions:**

These findings show that not all social influences were relevant within this setting.

**So What?:**

This study informs future research and interventions by indicating the social influences that exist within an Australian university institutional context, and therefore which may be used to change eating behaviour. Additionally, it provides evidence for the successful use of the CST as a theory to identify social influences and inform intervention development and planning.

## 1 Introduction

The transition from high school to university is a significant life event, involving increased autonomy, independence and often changes in living arrangements and social relationships. Evidence indicates university students have unhealthy lifestyle behaviours and high rates of health‐related risk factors [1]. Furthermore, students who reside on campus have been observed to gain weight during this period, often over a short timeframe (months) and faster than in the general population [2]. The eating and health behaviours established during this period are important determinants for ongoing health and the prevention of detrimental weight and obesity in adult life [3].

Eating behaviours are continuously shaped through social interactions between individuals and their environments [4]. Eating is recognised as complex and nuanced, further complicated by the lack of a consistent eating behaviour taxonomy [5]. Identified influences on eating behaviour include individual, psychological, physiological, environmental, cultural and social factors [6]. Importantly, social influences have been extensively shown to be a primary determinant of eating behaviour [4] and have been identified as potential mechanisms for successful eating behaviour change [7].

The American Psychological Association defines social influence as ‘any change in an individual's thoughts, feelings, or behaviours caused by other people, who may be actually present or whose presence is imagined, expected, or only implied’ [8]. Social influences, including modelling, social norms, reinforcement and interaction have been shown to affect dietary practices [7], indicating more needs to be done to translate research in this area to promote positive eating behaviour change [4]. Few studies have incorporated a solely social focused theory to guide research in this area.

In the study of eating behaviour, it is important to consider the environment in which eating takes place [9]. Institutions (e.g., health facilities, mining, military and educational) have been identified as promising settings for improving eating behaviour, as they provide physical access to a population and can allow for variable modification during interventions. Health promotion within settings or place‐based health promotion, views a setting, its people and its processes as a system with multiple intervention opportunities [10]. Place‐based research recognises that individually focussed health interventions may require high levels of individual agency to achieve health benefits, and differences in environment (or place) may prevent success or reinforce health inequalities. Calls have been made to understand the effectiveness of place‐based approaches that improve the physical, social and economic environment around people. Furthermore, the absence of social intervention strategies in place‐based research has been noted [11]. Institutions provide an appropriate setting for studying social eating behaviour, given the often‐communal dining environment [12]. Dining halls within university institutional accommodation can also be particularly social, compared to other settings (e.g., hospital), making them ideal for in‐depth exploration of social influences on eating behaviour. As university students are mostly young adults still developing their consumer eating behaviour [9], this setting provides additional advantages for studying the social influences on their behaviour. Despite extensive knowledge that eating behaviour is largely social [4], few Australian studies have explored the social factors influencing eating behaviour within an institutional setting where individuals select meals from a variety of provided dishes [13, 14].

The Consumer Socialisation Theory (CST) [15] describes how young individuals develop consumer‐related skills and behaviours through socialisation [16]. The CST states that social influences (specifically social interaction, social modelling, social norms and reinforcement) are social learning mechanisms delivered through socialisation agents (other people/mediums) [16] that influence an individual's consumer‐related outcomes and behaviour. For this reason, the CST is particularly relevant for studying how young adults develop food and eating behaviours and how these are impacted in response to social influence factors. Additionally, it emphasises the evolving process of learning consumer‐related behaviours over time in accordance with a changing social landscape [17]. The CST has been previously used to investigate topics such as online behaviour, product placement, food shopping and alcohol consumption [18, 19, 20, 21]. However, despite extensive evidence which supports the impact of the four social influences included in the CST, on behaviour [7, 22, 23], and its emphasis on both socialisation factors and consumer‐related behaviour, to date, no known studies have applied the CST to examine eating behaviour. While broader theories of eating behaviour incorporate a mix of biological, economical and psychological factors (e.g., The Health Belief Model [24] or The Theory of Planned Behaviour [25]), they introduce competing influences that dilute the social focus of this study. The CST allowed for clearer conclusions about the social drivers of eating to explore whether there is any value in applying theories that cover a range of social influences.

The aims of this study were twofold. First, this study aimed to identify which social influences from the CST (interaction, modelling, social norms and reinforcement) were evident in the eating behaviour of young adults within an Australian university setting. Second, this study aimed to explore whether the CST could be applied to gain insights that can inform an eating behaviour change intervention.

## 2 Methods

### 2.1 Participants and Recruitment

The study followed a qualitative research design using a convenience sample of university students who met inclusion criteria. The planned number of interviews was based on recommendations for reaching saturation of coded qualitative interview data (≥ 12) as determined by Guest et al. [26]. Participants (*n* = 16) were initially recruited using flyers displayed within one Queensland university live‐in college. Snowball sampling was used to enhance recruitment efforts through the referral of individuals by the initial study participants [27]. Inclusion criteria required participants to reside in an institutional university college that provided all three meals to residents (i.e., breakfast, lunch and dinner), with residents able to choose their preferred meal from a range of options. All participants' confirmed meals were included as part of their accommodation fees. Once contact was initiated, participants received an email containing a participant study information sheet, interview scheduling information, and a consent form. Upon completion of the interview, participants were offered a $50 voucher as a reimbursement for their participation.

### 2.2 Study Design

One‐on‐one, semi‐structured interviews lasting no longer than 60 min were conducted. The interviews were carried out by the moderator either face‐to‐face or via Microsoft Teams (Version 23320.3021.2567.4799). All in‐person interviews were held at a convenient location, walking distance from participant college accommodation. Interviews were recorded, de‐identified and transcribed verbatim, with note‐taking carried out by the moderator. NVivo (QSR International Pty Ltd. Version 12, 2018) software was utilised for coding and organisation of data. Interviewer's responses and filler words (e.g., ‘like’) were removed from the reported examples.

This study was conducted according to the Declaration of Helsinki, and all procedures involving human subjects were approved by the Griffith University Human Research Ethics Committee (GU HREC Ref: 2022/485). All participants provided written and verbal consent prior to data collection.

### 2.3 Applied Theory

**FIGURE 1:**
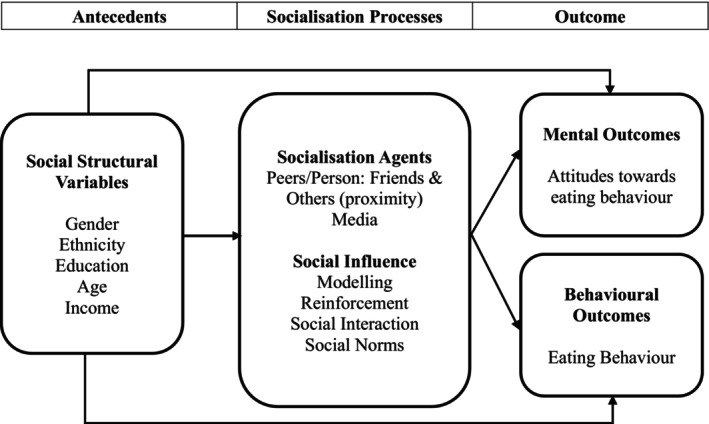
Proposed conceptual consumer socialisation theory (CST). The CST model was adapted for this study from de Gregorio and Sung [19].

The CST is comprised of four distinct categories: (1) antecedents; socialisation processes containing (2) social learning mechanisms (hereafter referred to as social influences e.g., modelling), and (3) socialisation agents (e.g., peers); and (4) outcomes (e.g., mental and behavioural). Figure 1 depicts the conceptual model of the CST applied in this study and adapted from that used by previous researchers [16, 19]. The extension to the CST theory was the inclusion of social norms as an additional social influence, given the evidence showing the influence of social norms on consumer (eating) behaviour [22].

### 2.4 Interview Guide

Interviews followed a semi‐structured format [28], with questions mapped onto the CST social influence constructs (examples in Table 1). Interview questions were designed to elicit open and natural responses aligned with the constructs, with verbal prompts included such as ‘*can you give me an example?*’, ‘*how?*’, or ‘*paint me a picture*’, to ensure participants elaborated on their replies. Minimal identifying information about the study topic was provided to participants to reduce bias. The interview questions were pilot tested with a member of the target group and adjusted accordingly where questions did not prompt responses that were related to the phenomena or constructs being examined.

**TABLE 1:** Examples of interview questions used to collect the CST social influences.

Social influence	Example 1	Example 2
Interaction	‘Do you think others notice what you eat? Who are they? What do they say? Do you think they notice but don't say anything?’	‘Do you eat differently when they are around? How and in what way?’
Modelling	‘Is there someone in your life who has inspired the way you eat or the things you eat? Who, and in what way?’	‘Who is your ‘food’ hero and why?’
Social norms	‘If you are in a new environment (e.g., work) how do you make the choice of what to eat at mealtime?’	‘What do you like to do while you eat? Does the meal or time of day impact this? Why?’
Reinforcement	‘Has anyone complimented or praised you, or given you indication that they are impressed with what you eat or what you cook?’	‘Can you recall any negative experiences with food? How does this influence you now?’

### 2.5 Coding Framework

Coding was based on the CST [15, 16, 19] (see Table 2). Coding guidelines focussed on the socialisation agents, social influences and outcomes. The most relevant social agents [36] for the present study were considered to be in‐person agents (e.g., university/college peers), as well as media agents including social media, online or mainstream media. Social agents were assumed to be relevant in an institutional setting (e.g., highest proximity to participants), and those most likely to be encountered by young adult university students. The social influences included were those named in the CST theory, and definitions for each of these influences were sourced from the literature to guide coding. Outcomes were mental (i.e., attitudes, beliefs and knowledge) or behaviours pertaining to consumer eating.

**TABLE 2:** Coding guidelines.

CST Construct	Citation	Description
**Socialisation Agents**		
*Peers/Person*		Peers/Others (expected close college proximity)
*Media*		Social‐media/Mainstream media
**Social Influences**		
*Interaction*	[16, 29]	May include a combination of social influences and refers to the way two or more people, groups or social systems mutually influence one another.
*Modelling*	[16, 30, 31]	Describes how individuals can learn behaviours through observation, and is the imitation of an ‘agent’ or another person's behaviour.
*Social Norms*	[22, 32]	Unwritten rules, beliefs, attitudes and behaviours that are considered common in social groups, culture, or society (i.e., perceived rules).
*Reinforcement*	[16, 19, 33, 34]	Strengthening of a specific response using a reward (+ reinforcement) or punishment (− reinforcement) to ‘reinforce’ a desired behaviour.
**Outcomes**		
*Mental*	[16, 19]	Attitudes/beliefs/knowledge: about food or eating behaviour.
*Behavioural*	[19, 35]	Eating Behaviours (the food choice, consumption, and regulation of eating, or the when, how, how much, what and why food is being consumed)

Social interaction was assumed to be present whenever two or more people, groups or social systems were mutually influencing one another, and therefore was not easily separated from other social influences like social norms, modelling and reinforcement. Observational learning or imitation of another person was coded as modelling. Social norms were coded to injunctive and descriptive norms [37]. Reinforcement (i.e., positive, negative and punishment) was defined as requiring an external and socially derived stimulus (e.g., peer praise/support). Intrinsic reinforcement (e.g., feelings of enjoyment, self‐praise, shame) was not coded as reinforcement for this analysis.

### 2.6 Data Analysis

A deductive content analysis method informed by the CST was conducted using NVivo Software and guided by the agreed definitions listed in table 2. Intra‐coder reliability [38] and inter‐coder reliability techniques were used. Duplicate coding was carried out on 25% of transcripts (*n* = 4), selected using a random number generator [39], until consensus was reached. All members of the research team discussed coding during analysis to reach agreement. Although mutual exclusivity between the four social influences within the CST was desired, this was not always achieved, particularly because social interaction often co‐occurred with other influences.

## 3 Results

Sixteen interviews were conducted over a span of 3 weeks. The total number of interview recordings (*n* = 16) equated to 11 h 31 min 17 s (*M* = 43 min 12 s) of audio. The longest interview lasted 1 h 04 min 13 s.

### 3.1 CST Socialisation Agents

Participants described instances of social influence being delivered through both the CST socialisation agents of peers and media (see Table 3). Peers were the in‐person socialisation agents most described and often included current friends from college who seemed to encompass different levels of health knowledge and experience. Other in‐person agents listed within college proximity included dining hall staff and other university students. However, these other agents were not described as often as friends and peers.

**TABLE 3:** Qualitative findings of the CST socialisation agents.

Social agent	Description	Example
*Peers* For example, friends from college, work and university	Peers (similar age, interests, background or status), or those in immediate environment [40]	‘*Yes. Definitely. I think the best way to get information is from your friends and your peers. That's your best source of information because they're people your age. It's good and bad because people your age don't necessarily know everything either. Hopefully people your age are well educated enough about the fact*’.
*Social‐media/Online* For example, social (e.g., TikTok) and online (e.g., YouTube)	Influences through online or mobile apps (e.g., TikTok, YouTube) [41, 42]	‘*I don't know if I have a particular one*. *I've seen a few videos by this dietitian online. And, I really like her because she talks about how much diet culture sucks. She'll watch TikTok's and review them and be like, “They're wrong. This isn't based on evidence,” or whatever. She breaks down a lot of the bad myths that are out there around food that I don't really like probably, yeah, because she is very sassy…YouTube and Instagram I think?*’
*Mainstream media* For example, documentaries or T.V. programs	Any other media source such as movies/shows, news, and magazines [43]	‘*I feel like during my life, different things. I went through my big vegetarian, vegan, minimalist, I‐want‐to‐live‐in‐a‐van stage life. I feel like those people on YouTube. I watch a lot of YouTube. And, they'd be like, “Mom of four living in Hawaii”. I'm just like, “Yeah. I want to”. I'm a big documentary person. I got a lot into the different food documentaries and the meat industry documentaries and all of that. So, I feel like that contributed to me going vegetarian and vegan and stuff*’

Agents operating through a media platform, such as an admired athlete or someone seen as having lived experience, were often described as being the participant's ‘food hero’, or someone they respected, aspired to be like, or follow the advice of. This admiration or aspiration was described in connection to media socialisation agents more often than through peer agents such as friends. Across all socialisation agents, those with expertise or perceived trustworthiness (e.g., with lived experience, close connection or with desirable qualities), were favourably described. This was especially evident within participants' descriptions of their acceptance and openness to engage in information sharing (e.g., food related knowledge), through social interaction and the likelihood of modelling eating behaviour:

If I know that that person is knowledgeable and trustworthy with that information. Obviously, with that information about the protein I took that with a pinch of salt because I don't know how reliable it is. For example, I would trust someone's opinion if they study nutrition or things like that.

Although not the focus of this study, other agents were mentioned and should be noted. These included work colleagues, coaches and family. Participants often expressed that family members such as primary caregivers, were still seen as their current ‘food hero’, and as agents still influencing their current eating behaviour.

### 3.2 Social Influences

Participants described the presence of all four CST social influences within the university institutional eating environment (Table 4).

**TABLE 4:** Qualitative findings of the CST social influences.

Social influence	Definition (see Table 2)	Example
*Interaction*	May include a combination of social influences and refers to the way two or more people, groups or social systems mutually influence one another.	‘*I'm at college, so we'll walk down together with the girls I live with. There's eight of us there, sometimes two, three or seven of us, we'll walk down together, stand in line, get lunch from college. There's a cafeteria and you pick what you want. I usually get salad and some meat and rice or something. Then, I'll sit down with those same people, plus whoever else is there. If there are people from another corridor that we're friends with, we sit with them and have lunch*’.
		‘*I love eating. I love the experience. I love sitting down with people having a meal. It's always been one of my favourite times of the day because we just chat about our days. You get to eat together. It's always something that I look forward to. Partly because of need, partly because I can just hang out and talk to people*’.
Modelling	Describes how individuals can learn behaviours through observation and is the imitation of an ‘agent’ or another person's behaviour.	‘*My mother's been vegetarian for the last 35 years. Because she's vegetarian a lot of meals I had back home were vegetarian, so I did get an appreciation for vegetarian options, so sometimes I do like to have that*’.
		‘*Well, I've actually started eating less meat because I've got a mate who's a vegetarian for all the environmental impact that the meat has. He just told me about it and told me to watch a documentary. So, I've been cutting down my meat intake and supplementing it with other protein sources*’.
*Social Norms*	Unwritten rules, beliefs, attitudes and behaviours that are considered common in social groups, culture, or society (i.e., perceived rules).	‘*In terms of the actual action of eating, I'm certainly more conscious of not making a mess or being a slob on myself. Especially if it's a messy meal, I'll feel bad for putting peas on the table, you know, being a slob or something…*’
		‘*Yeah, it's a communal eating area, and it is strongly encouraged for everyone to eat together. There's about 250 of us, and we have this big dining hall. Big tables that seat maybe eight to ten people. And then, basically, everyone lines up at 5:30—between then and maybe six o'clock. Everyone comes in and eats and sits down with their mates and chats. It's actually really nice*’.
Reinforcement	Strengthening of a specific response using a reward (+ reinforcement) or punishment (− reinforcement) to ‘reinforce’ a desired behaviour.	‘*I think I've been complimented on discipline. There's times where I've, you know, said no to things and stuff like that. I was really good that I can be disciplined when I need to be. Like, if I have something coming up where I know I need to cut back on certain things, like, wow, you're really self‐controlled. I feel like, overall, at college, we try not to talk about what people eat in general, like, whether it's positive or negative. Just ‘cause, you know, food is quite a sensitive topic*’.
		‘*The guys in my corridor often make fun of me for constantly ordering food, everyone's always trying to save money at college. I've noticed that I spent quite a bit last semester on just ordering food, so I'm trying to cut back a little bit but still do it, ‘cause it's better*’.

#### 3.2.1 Social Interaction

Social interactions were described by participants as occurring both singularly (e.g., chatting with friends over a meal) and concurrently with other social influences. For example, the positive reinforcement of praise could only occur when a participant has a social interaction with another person. Additionally, modelling was always described with social interaction, allowing for imitation of the modelled behaviour (e.g., number of salads consumed based on others). Social interactions were often indicated in participants' descriptions of their normal, everyday eating routines, like going to meals with those from their corridor at set mealtimes:

Lunchtime is usually, you know, talking or planning or everyone's just, you know, socialising. And, at dinner it's about the same. It's just talking and – and socialising a bit…

or through the reported exchange of information (e.g., what tastes good that day or nutritional knowledge):

I mean, we sometimes comment on each other's dinners, not in a harmful way, but just, like, you got the dumplings. Are they any good?

Social interactions differed depending on the preference of participants for what to do whilst eating, which included eating together vs. alone, talking with friends, and using mealtimes as a break or to ‘debrief’ with others.

Some mentioned social competition (e.g., going early to meals to ensure their favourite choices were not taken). In addition, some, but not all, participants mentioned making changes to eating based on certain social interactions. For example, choosing to eat with those they did not know at breakfast when friends were absent, or selecting meals to align with others' choices based on shared knowledge and information. However, cooperation was expressed by all participants and was seemingly a big part of their eating experience (e.g., sitting with others to avoid eating alone) and behaviour (e.g., eating differently as to not inconvenience the group).

#### 3.2.2 Code 2: Social Modelling

Most participants described modelling, and often in combination with social interaction (e.g., watching other students look after themselves through diet and exercise). Participants described modelling that occurred mostly through the live modelling of others (e.g., in‐person or live demonstration through media). This was particularly evident when a social connection was positive or intimate in nature (e.g., partner). Modelling was described by some as symbolic or as a desire to be like someone they may not directly know (e.g., athlete). Symbolic modelling, as with live modelling, was related to favourable perceptions of the socialisation agent (e.g., if a participant played sport, they may model the eating behaviours of their sports idol).

I don't know, she was, quite, like, a very fit, healthy, always looked really positive and someone I was like, wow, that'd be really great if I could be as, you know, be as fit and healthy and as you.

In contrast, verbal modelling was described as spoken instructions of what to eat or through description of the agent's eating behaviours. Moreover, verbal modelling was rarely mentioned independently from live modelling or originating from socialisation agents within the immediate environment (instead it originated from other agents such as family). All modelling appeared to be strongly connected with the participants perceptions of, and connection with the socialisation agent involved. The desirable qualities of the agent, and those that would be gained from the adopted behaviour (e.g., fit and/or healthy), were also important.

#### 3.2.3 Code 3: Social Norms

Injunctive norms, involving judgement towards one's own behaviour (e.g., appropriate quantity of food on a plate), were described as how others perceive their eating choices or behaviour. For example, not wanting to be seen as a ‘slob’ when eating around peers. Additionally, participants described norms as how they viewed or judged the eating behaviour of others. For example, the quantity of food taken in one serving, combination of food choices, taking entire servings for themselves or to share exclusively with their group of friends (e.g., dessert):

At college where I am now, the only time people notice what you're eating is if you have a weird looking meal. Or a really bare plate. Or something like, you've done a weird combination.

Descriptive norms were described as the eating behaviours they saw most others doing around them, and the subsequent desire to adapt their eating behaviour to fit these descriptive norms (e.g., increased eating speed if others were finishing faster). Participants also described what they saw others doing within the eating environment, such as noticing most students enjoyed eating together while chatting, especially at lunch and in the evenings when there was more perceived time. However, these descriptions were often subjective for the individual participant (e.g., people being uncomfortable to eat alone). Social norms were mentioned in a historical context (e.g., norms of childhood eating) and in the current environment (e.g., all students going down together for meals at mealtime):

And then, basically, everyone lines up at 5:30 – between then and maybe six o'clock. And, everyone comes in and eats – and sits down with their mates and chats. It's actually really nice.

#### 3.2.4 Code 4: Reinforcement

Reinforcement was described as the positive addition of a reward (e.g., praise for making disciplined or conscious food choices—vegetarian) or through displays of social support (encouragement of ‘good’ food choices) or to avoid negative or unhealthy thoughts or judgements (e.g., restrictive eating). Reinforcement through the removal of an influence or factor specific to the individual was also described. For example, the college eating environment appeared to reinforce certain eating behaviours for some participants through the removal of specific and identified others from the past who have judged or criticised eating behaviour, thus contributing to a reinforcing mechanism. In contrast, social reinforcement through punishment (e.g., criticism from others) tended to be detailed mostly in a historic context (e.g., criticism/judgement/punishing action in youth from sporting coaches, school peers or family), particularly surrounding weight and body image. Punishment was rarely included in participant descriptions of their current dining experience. However, when it was reported it was described as developing from differences between individual eating preferences like that of students who preferred to order in (e.g., Uber Eats), thus resulting in comments and teasing from others:

They'll just be like, “doing it again,” ‘cause every time you order something it's always got another bag with it. At the end of the week, I just come out with all my bags. They just watch me…

### 3.3 CST Outcomes

Participants described both the mental (e.g., believing a need to increase protein and therefore increasing intake) and behavioural outcomes (e.g., modelled increase of salad consumption) related to the CST (Table 5). Many of the behavioural outcomes recounted by participants indicated a subconscious, rather than conscious, awareness of the social influence impacting their eating behaviour. For example, some participants were unable to recount social influences in their current eating environment or provided limited details on the impact of others on their eating behaviour. However, some were able to identify mental outcomes associated with social influences:

I think because everyone was so aware of that (fresher five), personally I feel like I started making, well, not making, just being conscious of my food choices.

**TABLE 5:** Qualitative findings applied to the CST.

Social Processes	Outcome	Example
*Social learning/Influence* Social Interaction	*Mental* Views to connect. Views as a break from study.	‘*And so, often I'm working with my door shut. Then I'm stuck in my room all day, so it's lunchtime, let's go talk to someone. Or you might text the group to go, hey, who wants to go to lunch? And, then you can meet together and just talk or hang out and be with other people and socialise*’.
*Socialisation Agent* Peers	*Behavioural* Eat with others. Connection.	
*Social learning/Influence* Social Norms	*Mental* Beliefs about eating speed. Enjoy food less.	‘*I don't know. Probably, in terms of eating style, I always tend to eat quicker when I'm around people. I'll tend to be aware that I'm a slow eater. So, I'm going to enjoy the food less*…’.
*Socialisation Agent* Peers	*Behavioural* Eating more quickly.	
*Social learning/Influence* Modelling	*Mental* Beliefs about trying new things. Beliefs about a balanced/healthy diet.	‘*It's probably just been my parents for good, and healthy balanced diet. It's good. They support me in ventures. I, kind of credit them to—I'd always try the foods as a kid. There was always this thing which, “I don't care if you don't like it. Have you tried it? Try it. If you don't like it, that's good, come back to it in, like, two years or something”. ‘Cause I hated mushrooms up until two years ago and suddenly I love them, and my dad one day just fried them up with garlic, and I said, “Oh my god, that smells so good.” And, when I ate it. Then I tried it a year again and I was, like, “Oh my god, these are really good.” I have mushrooms, like, every day for breakfast, ‘cause they have that there now*’.
*Socialisation Agent* Others (family)	*Behavioural* Re‐trying food now offered in current eating environment.	
*Social learning/Influence* Reinforcement	*Mental* Beliefs of meal enjoyment. Attitude about having friends.	‘*I think for some people, maybe, eating in the dining room is a negative experience for them, ‘cause they don't have close friends. Like, maybe I wouldn't enjoy my meal as much if I wasn't really friends with anyone at college. I'm very grateful that I do have a close group and I find that, even if my friends aren't there, I'll try my best to sit at a table. Even if I don't really talk, I'll just listen. Like, sometimes you've just got to push yourself out of that comfort zone sometimes, and it is a bit awkward*’.
*Socialisation Agent* Peers	*Behavioural* Eats with others.	

Mental outcomes often resulted from judgements and beliefs towards others, (e.g., ‘surprise’ that others skip breakfast due to modelled importance growing up), self‐eating behaviour (e.g., believing it is important to try new things/foods) and beliefs and knowledge around nutrition and eating behaviour (e.g., viewing it as better to see food as sustaining/balanced, rather than being overly cautious). Furthermore, the behavioural and mental CST outcomes were often reported simultaneously. That is, they were not often described in separation from each other (e.g., beliefs or knowledge on what is ‘healthy’, leading to consumption choice). Participants in this study described all social processes and outcomes from the CST model in relation to their eating behaviour, in the studied institutional eating environment of a live‐in university college. Table 5 (above) summarises the representation of all the included theory elements detailed from the interviews with participants.

## 4 Discussion

This study was the first to examine the social influences evident in eating behaviour within an Australian university institutional eating environment using the CST. The findings confirm the presence of all four social influences of social interaction, modelling, social norms and reinforcement. However, reinforcement was referred to less than other social influences by this group. Additionally, the study was able to demonstrate the utility of the CST for exploring socially related eating behaviour within social marketing research. Although other studies have explored social influences on eating behaviour outcomes, few have explored multiple social influences at once within the institutional environment [44]. This study provides a unique and detailed investigation of these four social influences within this context, both simultaneously and individually, and makes multiple distinct contributions.

### 4.1 CST Socialisation Agents

The application of the CST identified important socialisation agents and associated attributes (e.g., expertise), required for social influences to be effective in this setting. This group did not consider media a common socialisation agent. Instead, they described the influence of in‐person agents as a main contributor towards socially influenced eating behaviour. When socialisation agents were mentioned, both media (or remote agents) and in‐person agents possessed desirable attributes such as expertise or trust. This is in contrast to others' findings of the influence that media (e.g., social media) has on young adult and adolescent health and eating behaviours [45]. However, this finding is supported by Higgs [22], who reviewed social norms research and found social norms are more likely to be followed when connected with a socially proximate group, especially when an intimate or close relationship was already established. Others have found modelling of eating only occurred when in‐group members were involved [46]. The current study aligns with these findings, showing that participants described favour towards a conscious or subconscious adoption of social influences when socialisation agents were considered social connections.

### 4.2 Social Influence

Congruent with previous research, the present study found social interaction was an important factor in the development and practice of eating behaviour [23]. This is due to the role interaction has in enabling other social influences (e.g., modelling), as well as in the everyday enjoyment expressed by participants to participate in mealtimes to interact, share and connect with others. Interactions were positively described and were largely connected with the facilitation of the sharing and exchanging of knowledge, support and an expressed desire towards cooperation over competition. Moreover, the positive interactions within this setting fostered feelings of safety, allowing participants to engage in preferred or newly adopted eating behaviours without judgement and with added social support. Participants indicated that social influence played a part in improving their relationship with food, which did not exist prior to their university experience. These findings indicate social interaction is a critical influence within this eating behaviour context, with positive interaction providing a potential pathway for positive eating behaviours.

Modelling was also an important influence on the eating behaviours of this university sample. Social modelling has been shown to have considerable influence on certain eating behaviours (e.g., food consumption), especially when combined with other social influences such as norms [7]. These concurrent social factors which facilitate or contribute to social modelling may explain the modelling examples described by participants within this study, especially when considering the communal dining setting (i.e., interplay of multiple social influences). Although some studies have found no difference between the likelihood to model the behaviour of a live or remote (e.g., video/social media) socialisation agent (or confederate), others contradict these findings and suggest shared social context may be more important [4]. This importance of shared context may give explanation for the present study finding a limited reporting of verbal and symbolic modelling.

Social norms have been extensively researched to understand and predict behaviour [47]. However, norms change over time, and differences between contexts may determine the strength of social norms [48]. This study has contributed knowledge of social norms in relation to consumer eating behaviour within the examined context and population. These findings include injunctive norms related to eating behaviour, developed through previous and present experiences of participants. Participants who had strongly held beliefs or judgements about eating behaviour referenced norms more often. This indicates the need for careful consideration of context for this influence and suggests that other contributing factors (e.g., experience) need to be explored in detail to better explain these individual differences and variability [22]. In contrast to the injunctive norms, are the descriptive norms which relate to what participants see others ‘normally’ do, which seemed to impact them mostly in the presence of those others (e.g., adjusting their diet in the norm specific environment). Although there is some debate about how long a social norm may influence behaviour, it is possible that this finding indicates a limited influence beyond the environment pertaining to the social norm [49]. Nevertheless, descriptive norms remained influential in the context of this study, especially when entering a new environment and prior to developing strong connections or friendships.

Reinforcement has been studied in both eating behaviour and behaviour change contexts. However, this study did not indicate reinforcement as an influence commonly experienced by these participants. Positive reinforcement, such as receiving a compliment for making healthy choices, was described more often in this cohort, which others have concluded is more effective at influencing eating behaviour [50]. Negative reinforcement was also described (e.g., teasing from others around ordering large amounts of takeaway food), which did seem to influence a change in behaviour. However, while participants could describe experiencing this influence, other scholars have found that the inclusion of negative reinforcement within interventions has not had the intended effect on behaviour as predicted [51].

### 4.3 CST Outcomes

The CST mental and behavioural outcome findings within this study highlight and contribute to the knowledge of what social influences are described most consistently with a particular outcome. One of the most distinct findings was the impact of participants conscious or subconscious awareness of the social influences on the reported outcomes consistent with their behaviour. For example, the level of awareness held by participants of the social influences impacting them not only determined the level of detail provided but also their specific eating behaviours and outcomes. When probing questions were used to elicit more social‐focused responses, it appeared to increase participant awareness around the social levers contributing to their eating behaviour. While some studies have found that people appear to be more aware of the social influences on their behaviour than previously believed [52], it is still something to be considered when exploring social constructs, as the level of awareness may impact the study objectives and be an additional influence in the behaviour itself. Future research should therefore continue to explore how to improve the exploration of social constructs through self‐report measures and responses, where awareness may vary. Additionally, expanding evidence on social influence awareness across varying contexts and populations may assist in better control for the impact on behaviour outcomes.

### 4.4 CST

This research has demonstrated that the CST was able to explore and explain the social influences present in eating behaviour within the institutional setting. The CST aligned well with the current study aims and the focus on clearly identified socialisation processes. These findings are consistent with others who have successfully applied various versions of the CST for the exploration of socially influenced consumer‐related behaviour, with reported benefits related to the theory's flexibility and adaptable design [19]. Ensuring theory is appropriately used, especially within research exploring complex phenomena such as eating behaviour, can contribute to improved communication, interpretation and reliability of findings [5, 53, 54]. Although the CST was deemed an appropriate theory to underpin this study, it is important to recognise the research had a tight focus on the socialisation process and the learning mechanisms involved, rather than expanding across the whole CST model. This tight focus enables future implications for eating behaviour change intervention development to suggest more clearly the useful socialisation details within a unique context, leading to a higher chance of success through a more tailored approach [55]. For example, the findings of this study suggest that in‐person agents, such as peers (over media), may contribute to greater success in encouraging eating behaviour change through the use of the social influences explored, such as modelling (e.g., healthy options), social interaction (e.g., to facilitate a desired quantity or choice) and social norms (e.g., to signal and shape food choice). Without this focus, interventions may risk steering towards using more general factors or other proven social influences not found present in the explored cohort. Therefore, consideration of which influences may be modified during intervention using a zoomed‐in social focus on the socialisation agents and influences, is important for tailored development towards successful future research and interventions on eating behaviour.

Others have examined social influence as a mechanism of action for eating behaviour change, reporting social influences as effective intervention levers [4, 7, 56]. The use of CST in this study indicates its utility within formative research, and the findings can inform the development of eating behaviour interventions using social influence in young adult populations. The findings suggest that this eating context (university institutional dining) could be an ideal setting to influence eating behaviour change due to several social influences being present, suggesting that they may be effective behavioural levers [56]. Interaction, modelling and social norms were described often in this university institutional eating environment. Socialisation agents (people) were more impactful when they possessed expertise or were trusted by individuals. This indicates careful consideration is required when considering socialisation agents and context. Social interactions that were positive in nature were linked with positive eating outcomes, suggesting that the tone of interaction created in intervention is important. Modelling may also be more effective when a thoughtfully chosen socialisation agent is used to improve, rather than reduce certain eating behaviours [4]. Given the insights generated in this study using the CST, this study demonstrates the suitability of the theory to identify the social influences that can then be built into healthy eating interventions. Therefore, it responds to the call for the advancement and improvement of theory within this field [54, 57].

### 4.5 Limitations

The present study had some limitations that should be acknowledged. A lack of consistent definitions within the literature limits conclusions drawn in this study to the definitions chosen. Future research could examine results arising from the selection of different definitions. As with all qualitative analysis, human error and subjective bias should be assumed. While duplicate coding aimed to increase reliability, it is recommended that future research continue to be conducted to reduce the likelihood of error.

This study is limited by the involvement of participants from a single university co in Australia. Given social influences are largely impacted by context, future research should explore social influences on eating behaviour in additional institutional settings, across changing eating landscapes, and with varying populations to deepen understanding. It is possible different contexts may provide different outcomes, helping to explain areas of low reference such as reinforcement, with the current study. Given family socialisation agents were described by participants, it would be beneficial for future research to incorporate family influences, despite participants now living in the peer‐dense college environment. Longitudinal studies are recommended to advance understanding of social influence on eating behaviour over time.

Eating is a complex set of behaviours with a widely established base of evidence demonstrating the myriad factors that influence the food choices people make. This study additionally focused on examining whether there is utility in considering a theory capable of disentangling the social influences explored. The CST was the focus for the present study, and four social influences were identified. Future research is recommended to understand whether a range of social influences can be identified using the CST across a variety of eating contexts using both qualitative and quantitative approaches. If the CST is supported across a range of studies, the social influences described by the CST can be included in other theoretical approaches (e.g., the COM‐B) to more effectively examine individual and environmental influences.

## 5 Conclusion

This study highlights the importance of exploring the complex social influences that exist within an institutional or communal eating environment. Findings indicate socialisation agents that possess desirable attributes, expertise or have established connections with the relevant population should be utilised to maximise behavioural change. Furthermore, modelling, social interaction and social norms were present in this setting, with reinforcement less so, signifying that the design of future interventions should establish a greater focus on these three social influences in forms that drive positive eating behaviour. Future research should continue to explore ways to utilise social influences and incorporate them as a mechanism to effectively promote eating behaviour change. The successful application of the CST with its specific social focus indicates that it can be used across other contexts to examine and inform intervention planning. These novel findings on the social influences found within the eating environment and behaviours of the young adults residing within an Australian institutional setting, help to inform future eating behaviour research and interventions that aim to increase the prevalence of healthy lifestyle habits.

## Author Contributions

**Katrina A. Pitman:** conceptualization and design, formal analysis, investigation, methodology, data curation, roles/writing – original draft, writing – review and editing, project administration. **A/Prof. Julia Carins:** conceptualization and design, formal analysis, methodology, data curation, roles/writing – original draft, writing – review and editing, supervision, project administration, funding acquisition. **Prof. Sharyn Rundle‐Thiele:** conceptualization and design, roles/writing – original draft, writing – review and editing, supervision, funding acquisition. **Dr Lisa Vincze:** conceptualization and design, roles/writing – original draft, writing – review and editing, supervision.

## Ethics Statement

This study was conducted according to the Declaration of Helsinki and all procedures involving human subjects were approved by the Griffith University Human Research Ethics Committee (GU HREC Ref: 2022/485). All participants provided written and verbal consent prior to data collection.

## Conflicts of Interest

The authors declare no conflicts of interest.

## Data Availability

Interview participants were assured raw data would remain confidential and would not be shared. Data not available/the data that has been used is confidential.
